# Can you be a peer if you don’t share the same health or social conditions? A qualitative study on peer integration in a primary care setting

**DOI:** 10.1186/s12875-024-02548-5

**Published:** 2024-08-12

**Authors:** Émilie Lessard, Nadia O’Brien, Andreea-Catalina Panaite, Marie Leclaire, Geneviève Castonguay, Ghislaine Rouly, Antoine Boivin

**Affiliations:** 1 Canada Research Chair in Partnership with Patients and Communities, Centre de recherche du centre hospitalier de l’Université de Montréal (CRCHUM), 850 St-Denis Street, Montréal, Québec H2X 0A9 Canada; 2 Public Health Agency of Canada, 200 René-Lévesque West Blvd, #102, Montréal, Québec H2Z 1X4 Canada; 3 Faculty of Medicine, Department of Family Medicine and Emergency Medicine, Université de Montréal, 2900 Édouard-Montpetit Boulevard, Montréal, Québec H3T 1J4 Canada

**Keywords:** Healthcare services, Implementation, Integrated community care, Primary care, Peer support, Social care

## Abstract

**Background:**

Peer support has been extensively studied in specific areas of community-based primary care such as mental health, substance use, HIV, homelessness, and Indigenous health. These programs are often built on the assumption that peers must share similar social identities or lived experiences of disease to be effective. However, it remains unclear how peers can be integrated in general primary care setting that serves people with a diversity of health conditions and social backgrounds.

**Methods:**

A participatory qualitative study was conducted between 2020 and 2022 to explore the feasibility, acceptability, and perceived effects of the integration of a peer support worker in a primary care setting in Montreal, Canada. A thematic analysis was performed based on semi-structured interviews (*n* = 18) with patients, relatives, clinicians, and a peer support worker.

**Findings:**

Findings show that peers connect with patients through sharing their own hardships and how they overcame them, rather than sharing similar health or social conditions. Peers provide social support and coaching beyond the care trajectory and link identified needs with available resources in the community, bridging the gap between health and social care. Primary care clinicians benefit from peer support work, as it helps overcome therapeutic impasses and facilitates communication of patient needs. However, integrating a peer into a primary care team can be challenging due to clinicians’ understanding of the nature and limits of peer support work, financial compensation, and the absence of a formal status within healthcare system.

**Conclusion:**

Our results show that to establish a relationship of trust, a peer does not need to share similar health or social conditions. Instead, they leverage their experiential knowledge, strengths, and abilities to create meaningful relationships and reliable connections that bridge the gap between health and social care. This, in turn, instills patients with hope for a better life, empowers them to take an active role in their own care, and helps them achieve life goals beyond healthcare. Finally, integrating peers in primary care contributes in overcoming obstacles to prevention and care, reduce distrust of institutions, prioritize needs, and help patients navigate the complexities of healthcare services.

## Background

The rise in the prevalence of chronic diseases and comorbidity poses a significant challenge to healthcare systems worldwide [1]. Similarly, poverty and global inequalities continue to worsen and have been exacerbated by the COVID-19 pandemic [2]. The complexity of medical and social problems (e.g., chronic pain, social isolation, poverty, depression, and anxiety), which mutually reinforce one another, are often tied to social determinants of health that require thinking outside of the box of primary care approaches. New models of primary care targeting social determinants of health need to be developed and evaluated to gain knowledge on the features, characteristics, and effects of diverse peer support practices in primary care. This article presents the results of a participatory research project on the integration of a peer into a primary care team in Montreal, Canada.

### Integrating peers in primary care teams

Peers are people with significant lived experience of a health condition (e.g., mental or physical disease) who have experienced social challenges (e.g., housing insecurity, homelessness, racism, and discrimination) or share social identities (e.g., Indigenous peers). Peers mobilize specific strengths (e.g., relationship building, listening without judgment, resilience, pragmatic goal setting, system navigation) and draw on their experiential knowledge to establish trustworthy relationships, reduce stigma and shame, offer hope, and build bridges between people, healthcare teams, and communities [3–11].

Peer support is anchored in empowerment models of care, reinforcing people’s strengths and abilities [5]. Peers “walk alongside” people experiencing health or social challenges, working towards their own goals and priorities, at their own pace: an approach that is fundamentally different yet complementary to that of healthcare professionals [3, 5, 12, 13]. Existing research suggests that peers improve individual care (e.g., quality of life and social support) [8, 9, 12, 14–17] and providers’ well-being (e.g., team cohesion and mutual support) [18–20]. Peers can also positively influence social determinants of health (e.g., housing and harm reduction) [21, 22] and lower healthcare costs (e.g., fewer emergency room visits) [23, 24]. Peer support is particularly effective with people marginalized by the healthcare system [25], helping to address barriers to health equity.

Despite these demonstrated benefits, peers can experience discrimination and often lack recognition from colleagues due to their lived experience of addiction, poverty, mental illness, or chronic diseases [8, 26, 27]. Peer support also entails unique challenges, including disclosure of personal experiences, being confronted with past traumas, and managing relationship boundaries [8, 9, 28, 29]. In North America, peers often lack basic institutional support [26, 29–31], which increases the risk of burnout and threatens teams’ sustainability [26, 31, 32]. Many jurisdictions do not recognize formal peer status, creating barriers to financial compensation and program scale [8, 26].

Peers are recognized as essential in health education, health promotion, and community health, and may work in various community or healthcare settings. One of the demonstrated impacts of peers is through their integration into care teams. Members of care teams can include different practitioners (e.g., nurses, social workers, outreach street workers, psychologists, physicians, community workers). While professionals tend to bring “vertical” expertise to the team (e.g., in pharmacology, psychotherapy, or social interventions), peers offer a “horizontal” integrative view of lived experience with the disease or social challenges (e.g., how complex healthcare system navigation can act as a barrier to treatment). Peers improve access and continuity of care across health and community sectors (e.g., linking primary care with community resources) [10, 22, 25, 33–37]. They can also support coordination with other sectors (e.g., the justice system or employment services). Peers can help reframe the role of people experiencing illness from passive recipients of care to partners in care: empowering them to undertake a proactive role and facilitating relationships with primary care team members based on their strengths and abilities [22, 38–40]. Peers tackle key barriers to prevention and care: mistrust of institutions, complexities in navigating services, and the need to prioritize when faced with overlapping issues [25, 41, 42].

Despite strong evidence, peer integration in primary care teams remains limited. While peer support interventions have been studied for decades in the fields of mental health, addiction, and other specific communities (e.g., homelessness, HIV, migrants, and Indigenous people), it remains unclear how they can be implemented in a general primary care setting that supports people with a diversity of health conditions and social backgrounds. The challenges and opportunities for peer integration into general primary care practices - where peers are not solely “matched” based on similar disease experiences or social identities - have rarely been studied. As part of a larger participatory research program (Caring Community), we studied the feasibility of integrating a peer into a primary care team by examining the role and its resulting practices, perceived effects, and ongoing challenges.

### Caring community participatory research program

**Fig. 1:**
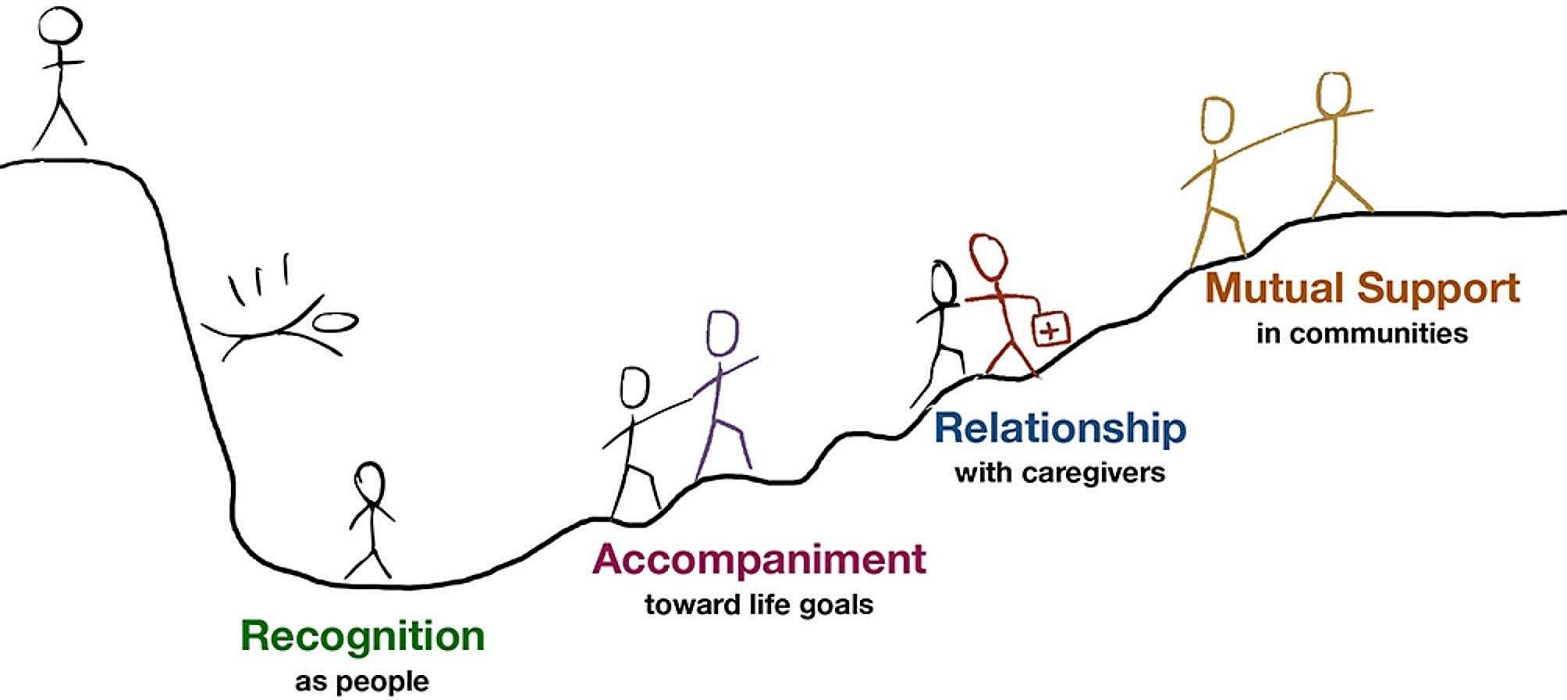
Caring Community model

Started in 2016, Caring Community is a participatory research program on the integration of peers in community care teams. Caring Community is grounded in principles of partnership and is co-led with peers and their healthcare partners. It takes a collaborative approach which recognizes the knowledge of all: peers, researchers, clinicians, community groups, citizens, and system leaders. While most peer support programs are disease or population-specific, Caring Community distinguishes itself by its integrated care orientation, as it serves people with complex health and social needs. The idea lies in the continuity of care between primary and community care, which aims to act on social determinants of health by increasing social capital, social networks, health literacy, and access to resources. The Caring Community provides a mechanism for cross-sectoral collaboration with the goal of promoting health equity by tackling social determinants of health through the development of a collaborative model of care between a primary care clinic, community organizations, citizens, and civic institutions. Caring Community offers a bridge between communities and healthcare systems, through the integration of peers in care teams. Peer’s role focus on: (1) Recognition of patients as people with agency, strengths and capacities; (2) Accompaniment of people toward their own life goals; (3) Fostering relationships with health and community ressources; (4) Supporting mutual support in communities (Fig. 1).

The vision of a primary care professional and a peer support worker caring together stems from the premise of the complementarity between professional knowledge of medical conditions (e.g., diagnosis, treatment options, and delivery) and experiential knowledge of living with a health or social condition (lived experiences of homelessness or being a patient navigating the healthcare system). The peer (GR) who has been integrated into the primary care team has a unique and outstanding experience that provides a skill set that is difficult to acquire. Not only has she been a patient since birth, but she has over 50 years of experience in accompaniment and over 10 years as a patient-partner in research, teaching, healthcare management, and direct care.

The peer’s contributions are not limited to direct care, such as patient follow-up. In the Caring Community, the peer also links health and social care and participates in the primary care multidisciplinary team meetings, where patients’ cases are discussed, thus contributing to the education and training of healthcare professionals. The Caring Community aims for integrated care for a population living in a historically underserved neighborhood in Montreal, Canada.

## Methods

To explore the feasibility, acceptability, and perceived effects of integrating a peer support worker into a primary care clinic, a participatory qualitative single case study focusing on peer support in primary care setting was conducted between 2020 and 2022. The aim of this paper is to identify the characteristics of peer support in a primary care and the distinctive features of integrating a peer in patient care without relying on disease or social identity criteria.

### Research setting

Our study was conducted in a Montreal (Canada) primary care clinic serving approximately 12,000 individuals in a community experiencing health disparities. The multidisciplinary team includes clinicians from different professional backgrounds: doctors, psychologists, nurses, social workers, managers, and a peer. In order to preserve the confidentiality of the research participants, we have chosen not to divulge the location of the study.

### Participatory research approach

Our research approach is oriented by the principles of community-based participatory action research (CBPAR), defined by Wallenstein et al. [43] as “collaborative efforts among community, academic, and other stakeholders who gather and use research and data to build on the strengths and priorities of the community for multilevel strategies to improve health and social equity.” CBPAR orientations imply that research not only seeks to produce knowledge with community members and stakeholders but also to bring about social change. Thus, the research team includes the peer (GR), clinicians (AB and ML), and other researchers/research team members (NOB, EL, ACP and GC). The participatory research approach means that some of the actors who were directly involved in the implementation of the Caring Community (e.g. clinicians [AB and ML] and peer [GR]) are also integrated into the research team. The peer hence participates in this study in a dual role: as both an implementer of the peer support intervention and a co-researcher, contributing to the study’s design and the interpretation of results. Other members of the research team act as outsider researchers who have conducted the interviews [NOB] and primary data analysis [EL and ACP].

### Participant recruitment and data collection

Purposive sampling was used to select and recruit participants (*n* = 18) for semi-structured interviews. Participants were patients (*n* = 6), patients’ relatives (*n* = 2) and primary care clinicians (*n* = 10). The inclusion criterion for patients and their relatives (*n* = 8) was to have had a joint follow-up by the peer and a primary care clinician between 2017 and 2020. The inclusion criterion for primary care clinicians (*n* = 10) was to have been involved in the larger participatory research project in the primary care setting between 2017 and 2020.

Due to pandemic restrictions, interviews were conducted online by Zoom or by phone between August 2020 and March 2021. Interviews were conducted by NOB in French (*n* = 17/18), and one was conducted in English. All interviews were transcribed verbatim and the names of all participants were anonymized and pseudonyms attributed. Cited quotations in French were translated to English by EL.

### Data analysis

Data analysis was grounded in a constructivist approach, which positions meaning and experiences as socially produced and reproduced [44]. We make the distinction between disease, understood as a biological condition, and illness, as a socially constructed meaning of the biological condition [45]. Guided by the six phases described by Braun & Clarke [46] (data familiarization, generating codes, searching for themes, reviewing and defining themes, and reporting findings), a thematic analysis was performed using QSR NVivo 12. Three themes (each including one or more sub-themes) reflect the research results on integrating a peer support worker in a primary care setting. We took care to compare the points of view of the patients and their relatives (*n* = 8) with the primary care providers (*n* = 10) to ensure the internal validity of the final results. Preliminary findings were discussed with the primary care team in the Summer 2022. Finally, results were discussed with the peer and the primary care clinicians to contextualize the findings, gain new insights, deepen the meaning, and bring nuances and context to the interpretation of the results.

## Results

Findings contribute to a better understanding of the roles, identity, mechanisms, characteristics, as well as perceived effects, risks, and challenges of the integration of a peer support worker in a primary care setting. Three themes encompass the study results: (1) Peer support intervention in primary care practices (roles, identity, mechanisms); (2) Perceived effects for patients and primary care clinicians (perceived benefits, effects, impacts); (3) Challenges and ethical considerations (perceived risks and barriers). Findings offer insights into how integrating peers in primary care settings can support people with a variety of health conditions and social backgrounds.

### Who finds support? Identifying people who could benefits from peer support in primary care

The Caring Community was initially implemented in a primary care setting in 2016 with the integration of a peer into the multidisciplinary clinician team. According to participants, the role of the peer was to provide social support and coaching beyond the care trajectory. Our findings showed that the capacity of the peer to fully play their role depended on the clinician’s understanding of the nature of the support offered, the available resources, the acceptance of the peer within the primary care team, and the actors involved in the project.

Clinicians reported making a referral to the peer when they felt they reached a therapeutic impasse (e.g., difficulty in gaining trust or creating a therapeutic alliance, a patient who does not show up for medical appointments), or when faced with important psychosocial issues and needs (e.g., poverty, social isolation, exclusion or violence), which require a high level of intensity in terms of accompaniment (i.e., duration and frequency of follow-up). When asked to describe the type of patients they referred to the peer, clinicians portrayed people with recurrent complaints, somatization (i.e., pain), non-medical causes of health problems (e.g., stress, poverty), or those for whom medicine can do little. The following quote illustrates the profile of patients referred to the peer from a clinician’s perspective:

“*One patient is very old*,* very isolated*,* but she somatizes a lot. So I had to dig around. Are there things that we could do as a team*,* or put in place with her living environment or her family to prevent everything from passing through us? It always manifests itself by complaints of pain*,* dizziness*,* anxiety*,* high blood pressure attacks. Now*,* it’s basically anxiety. Try a little bit of… I think* [the peer] *can help us to find the source of the problem with the patient*,* make them think about other issues. That’s one of the cases where there’s a lot of somatization*,* a lot of requests for help for the medical team*,* when we*,* euh*,* we can’t offer much. It doesn’t go through the medical for us.*” (Vanessa, primary care clinician).

Indeed, we find profiles with similar life courses, health problems, and psychosocial needs: aging, chronic pain, comorbidities, emotional issues, mental health (e.g., anxiety, depression, and post-traumatic stress disorder), poverty, and social isolation.

*Peer support as a listening space: taking time to nurture trustful relationships* According to clinicians and patients, once the referral to the peer was made, usually by a clinician in our study (e.g., a general practitioner, psychologist, or social worker), an initial meeting with the peer would take place in a primary care office. In order to create a bond of trust, the peer would introduce herself by adapting what she discloses or not according to the needs of the person being supported. The peer explained that she introduces herself as an accompanying peer who is part of the primary care team. As a trusting relationship is initiated and deepens over time, meetings can be held in alternative locations based on individual needs and preferences (e.g., community organizations or at home). Typically, the meeting with the peer is for a weekly period of about 60 min, but the intensity of the follow-ups varies from person to person. There is no single model, as the frequency varies according to the needs of the patient and the availability of the peer. For Elodie, a patient, the peer must “Keep in touch at least once a week at first, then you’ll have to wait and see. It depends on the person’s situation.” When we asked patients and their relatives to describe the frequency of follow-up, their responses showed that they associate the frequency of follow-up with the consistency of the support: being available when people need it, taking the time to listen to the person, and accompanying them on their journey, rather than meeting on a precise frequency such as weekly, bi-monthly, or monthly.

In addition, temporality is an important feature of peer support work, according to patients, clinicians, and the peer. The frequency of follow-up is related to the time it takes to establish trust and develop the relationship. Taking the time to listen and be a regular presence in the life of the person supported are necessary to generate bonds of trust that allow the peer to identify and meet their needs. Caroline, a primary care clinician, perceived the integration of a peer into the primary care team as an added value: “*I find that patients*,* especially those with distress or crisis*,* or even major physical health issues*,* they need to be listened to. I think the great added value of* [the peer] *is that she has the time*,* and then she takes the time to listen to those people.*” Some primary care clinicians said that time is a scarce resource, as they do not have time to truly listen to their patients to create a strong therapeutic alliance. The patients followed by the peer also mention the importance of time, not only to build trust, but also to develop a relationship:

“[*Doctors] don’t have the time*,* they have this or that*,* but it takes time (…) I’ve found that the most time-consuming part is developing a relationship with patients (…) Often*,* we [patients] keep things hidden and you have to go and get them*,* but you have to first get into a relationship to go and get them.”* France.

*Beyond shared experience: Peers as community connectors to bridge health and social care*Primary care clinicians describe the initial intervention model as related to the roles of the peer in the primary care setting: a social support and a coaching role that generates social bonds focused on peer support towards the caring trajectory. The participants describe the role of the peer in the primary care setting as holistic accompaniment and comprehensive support throughout the caring trajectory. According to the common understanding of all types of participants, peer support in primary care is characterized by an accompaniment based on active listening, unconditional acceptance, respect, non-judgment, and a vision that humanizes patients, each of which generates a bond of trust. As mentioned earlier, taking the time to listen without judgment and having a holistic vision of the person helps create trustworthy relationships. Once established, trust-based relationships create a safe space that leads the patient to become more aware of the problems experienced, to express their emotions and thoughts, and to share what they are going through with the peer.

The peer’s role is also perceived as a bridge between health and social care, as it helps navigate between the healthcare system and community resources. Patients and their relatives shared that the peer has a role of emotional, social, practical, and material support, serving as an advocate and guide when navigating the healthcare system and community resources. According to clinicians, patients, and their relatives, the most important feature of the peer’s work in the context of primary care is the ability to link identified needs with available resources in the community. As mentioned by Vanessa, a primary care clinician: “*Yes*,* there is the clinic*,* but after that*,* there is their community. It’s getting them back into their community*,* the community organizations*,* their family*,* their friends and the* [peer] *who is there as a kind of transition in between.*” Yan, a patient’s relative explains how the peer connects unmet needs with community resources: “*[My loved one] had needs*,* like at a certain point*,* housework is a very simple thing to do*,* but it’s something she can’t necessarily do. [The peer] could take actions that my [loved one] didn’t know anything about. I know that [the peer] knew a lot of resources that could really help.*” The peer’s work is therefore relative to its ability to navigate the healthcare system and connect needs to available resources in the community.

**Finding shared humanity in hardship: cultivating hope and empowerment as a catalyst for change** Findings about the perceived benefits and effects allow us to describe how the mechanism of the peer support intervention produced outcomes in the context of primary care. Micheline perceived their relationship as: “*Well*,* it’s as therapeutic as anything else. Being validated*,* finding trust and acceptance*,* non-judgmental*,* that*,* that is absolutely… That is key (…) I’m as comfortable as I can be*,* I accept the growing pains.*” By being receptive this way, the peer initiates a social recognition of suffering and validates painful experiences and emotions. Therefore, peer support work is perceived by the overall participants as a humanist, empathetic, and holistic approach to support, marked by openness, listening skills, patience, and wisdom.

Understanding and sharing experiences of the disease or hardship (recognition of suffering and validation of the experience) in turn promote ownership for living with a disease and/or traumatic experiences (e.g., pain, functional limitations, end-of-life, grieving, or sexual abuse). Louise, a woman having multiple chronic conditions while living in a precarious financial situation, explained:

*It helps me to understand a little bit my diseases because I have several* [comorbidities] *heart*,* diabetes*,* kidneys*,* anemia […] I am someone who doesn’t care about anything*,* but I know that I must care about my diseases*,* because if I don’t care about it*,* then I can die. I don’t accept it. Sometimes*, [the peer] *would tell me “I know*,* it’s not easy but you’ll get through it*,* think about your grandchildren*,* think about your children. It will help you”. [The peer] would motivate me a little bit*,* she would give me a little bit of courage.*

Sharing experiences is not based on similar social or health conditions, but, rather, on having gone through difficult hardships and having found ways to overcome these difficulties:

“*Because from the get-go*,* she shared some information*,* personal information about her health and her life and stuff. So although we don’t have exactly the same health problems*,* I know she also has had some more deals and experiences and*,* you know*,* because it is difficult trying to. Trying to get through the process of getting a diagnosis or an evaluation*,* so*,* you know*,* from what she shared with me*,* I knew that she wasn’t just blowing smoke*,* she knew.*” Micheline.

At the heart of the peer-patient relationship is a connection that fosters trust-building through understanding difficult experiences and hardships. From the peer’s point of view, overcoming hardship enables the peer to connect and build a relationship of trust, which is established by “finding ourselves in our common humanity.”

The peer support approach allows for the identification and fulfillment of essential needs that link health and social care (e.g., healthiness of the dwelling, access to food and health care, social connections, and a sense of security), which is often a prerequisite for feeling better. For example, after Louise shared that the peer has saved her life several times, the researcher asks to specify what aspect of the peer’s support feels the most important. Louise responded: “*Everything! The food*,* the support*,* the listening.*” Genuine and global concern for the other is the basis of the relationship. Understanding the experiences of the disease and hardship is therefore at the heart of the intervention mechanism because it makes it possible to establish bonds of trust and a sense of connection that leads the persons being followed to open up to the other, resulting in their feeling trusted, recognized as a full-fledged person, and validated in their experience. Louise explains how feeling understood gives her hope to learn to live with the disease: “*She told me that she understood me and that you can get through it even if you are sick. You can’t be totally cured*,* but if you are careful*,* you can have the same beautiful life*,* despite the disease.*” In doing so, the peer also initiates and supports meaning-making through shared understanding of their experiences of illness, hardship, and adversity, even though they are not similar.

As explained by Monique (a patient), when peers mobilize their experiential knowledge, they enable a greater awareness of the difficulties experienced by others and their ability to act on them:

“*She gives me the ammunition to act psychologically. She gives me tools and strength*,* and I draw on her strength to feel strong too. That’s why I admire those people who are able to talk to people like me*,* who have experienced hardship*,* who have been through stuff and are still going through it*,* and who have the patience to listen because you have to be patient to listen to someone in need.*” Monique.

As a result, changes in attitude (perception of oneself and one’s condition) give rise to hope for a better life despite the disease or hardships, which in turn leads to behavior changes (ability to act to improve one’s situation) and even emulation (desire to help in turn). As Elodie shared, “*Well*,* my confidence came back to me. Yes*,* I have more confidence in myself and I have more*,* I feel less weak than before*,* less weak from the point of view of the spirit. For example*,* well*,* I recognize that I have abilities. Good abilities*,* many things. I recognize myself.*” The desire to help in turn was a reason mentioned by most patients to justify their participation in the research. They consider that what they received from the peer should be given back when possible.

Primary care clinicians explained that they see the peer’s role as empowering patients to strengthen their involvement in their care: “*Patients can be coached by* [the peer] *to raise their questions with us*,* to speak frankly about what they are experiencing*” (Marie-France). Another clinician added, “*These are people with chronic illnesses*,* who I think need a little empowerment in their role as patients*,* in the system*,* for their appointments*,* for their needs*,* for their requests*,* even psychosocial ones”* (Evelyne). In addition, a primary care clinician mentioned that changes in attitude and behavior can lead to getting “*out of this position of being on hold or dependent on the health care system*,* to really say how ‘I take ownership of this pain*,* of my treatments’* ” (Maude). The mechanism of the aforementioned perceived effects is related to the ability to have confidence in oneself and in others, which makes it possible to hope for a better future, set life goals, have the courage to take action by making changes (even small). Setting limits and regaining control over one’s life through recognition and validation of one’s experience breaks the vicious circle of suffering and unhappiness, which could be transformed into an empowering quest for meaning to make sense of the illness or hardship.

*Perceived effects by primary care clinicians: understanding*,* trust*,* communication and collaboration*From a primary care clinician’s perspective, a peer integrated into their team was perceived as a reassuring presence: “*I find it reassuring to know that there will be a* [peer] *who will be there to listen to your patient*” (Caroline). By taking time to truly listen and gain trust, the peer helped to better identify the patient’s needs, facilitating collaboration between primary care clinicians and patients: “*The bond of trust was able to be established*,* so the person was more cooperative and it just improved her health and it helps the care providers for sure*” (Vanessa). For some clinicians, gaining knowledge of the patient’s needs and the issues they face was the greatest perceived benefit: “*I think the biggest benefit is that you end up understanding a little more about the patient’s issues and then their needs. It’s more named. We manage to have an agenda and a kind of common work plan*” (Chantal). Moreover, the peer also facilitated communication of the needs and issues between patients and their primary care provider: “*As a clinician*,* sometimes patients feel less comfortable opening up*,* talking about things. If the patient agrees*,* the peer will share with the care provider and they will exchange information and discuss the case. And that can move things along a little faster*” (Caroline). Since the initial referral to the peer stemmed from the need to overcome a therapeutic impasse, peer support intervention in primary care is thus perceived as helpful for overcoming therapeutic impasse.

Moreover, the peer’s experiential knowledge of the healthcare system enabled primary care clinicians to draw a clearer line between the roles and responsibilities of the various healthcare professionals involved in the follow-up of patients with complex health and social conditions. The integration of the peer in the primary care team is thus perceived by some clinicians as a means to expose the unique characteristics of their respective professional roles: “*They managed to navigate through a system. They understood each other’s role. They put us in front of each other*,* they are very good at revealing professional identities*” (Maude). The peer acts as an agent of socialization between diverse professional identities and contributes to the understanding of how the healthcare system works from a patient perspective.

Finally, primary care clinicians noticed a decrease in mistrust toward the health and social services system and clinicians, which is attributed to the role of the peer as a bridge between the health and community sectors: “*Often*, [the peer] *was the intermediary who brought a social worker or a psychologist or a community organization into the patient’s follow-up*,* in situations where I was very much stuck in the* [clinician]*-patient* [relationship], *as if the* [clinician] *embodied the answer to all the needs’’* (Sylvain). They also noticed that peer support allows them to get a more comprehensive view of their patients: “*Instead of just focusing on diabetes*,* we’re going to be more holistic (…) What is the quality of life? What are the medications? Are you really taking them? There are discussions that are maybe more honest*” (Chantal). A greater confidence in the health and social services system and a more comprehensive understanding of patients’ needs by health care professionals are linked to better quality of care, according to primary care clinicians.

### Uncharted Territory: ethical challenges and role ambiguity in integrating a peer support worker into a primary care team

Primary care clinicians initially had difficulty understanding the nature and limits of peer support work: “*What does a* [peer] *do when caring for someone? What don’t they do*?” (Hugo). Concerns also stem from clinicians’difficulty in understanding and delineating the boundaries of the peer’s role in the primary care team: “*There was the issue of really understanding what her role is. It’s an accompaniment*,* but how far does it go?* (…) *Because the role is not yet very clear*,* I think that this can create a kind of reluctance to refer*” (Chantal). These results illustrate the initial clinicians’ resistance due to the unknown limits of the peer support work, which was related to an anticipatory fear of unintentionally harming patients, fear of the unknown, and fear of exhausting the peer.

In the Caring Community research program, the peer provided support as a volunteer, but was compensated for her work within the research project. However, the absence of formal status within Quebec’s public healthcare system was seen as a major barrier to financial compensation of peers.

The peer must navigate between the flexibility of their role and the absence of guidelines for peer support practices within the context of a primary care setting: “*The fact that she is not obliged to be constrained in a frame of reference (…) when you enter an institution*,* there is too many structure*,* you are not allowed to do anything*” (Caroline). In addition, primary care providers expressed concerns about the personal limits in the relationship between the peer and those being followed: “[I]*s* [the peer] *able to set her boundaries as well. You don’t want it to interfere with the* [the peer’s] *personal life. Someone who starts calling her 8 times a day or sending her nonsense because she hasn’t called them back*? *(…) They don’t have a professional code of ethics*” (Chantal). In response to these concerns (e.g., financial compensation, absence of a formal status and limits of peer support work), an ethical reference framework to guide peer support workers in primary care is currently being developed by the peer and an ethicist.

## Discussion

The findings of this study demonstrate the feasibility of integrating a peer in a primary care setting without being matched on similar diseases or social backgrounds. Our findings show that the relationship between the peer and the people being accompanied is not built on a shared experience of a specific illness or living conditions but rather on having experienced medical and social hardships. Since the peer is a person who has managed to overcome hardships, the goal of peer support work is to help others do the same.

Our sample of people followed by the peer (*n* = 8) share similar socio-demographic characteristics (e.g., age, gender, and socioeconomic status). Given the small number of participants, we recognize that similar characteristics may not be in play. However, in our study, complex social and health issues are common to each person referred to the peer support worker. Thus, integrating a peer in this context has the potential to act on social determinants of health by linking health and social care in a meaningful way. Our findings are consistent with the literature on peers in mental health, addiction, and homelessness, as they tackle barriers to prevention and care, decreasing mistrust of institutions, prioritizing needs, and navigating through the complexities of healthcare services [3–11, 22, 25, 27, 33, 34, 37, 47].

Results also demonstrated the perceived effects and benefits for both patients and primary care clinicians, as well as the challenges and ethical considerations of integrating a peer into a primary care team. By mobilizing experiential knowledge, strengths, and skills, the peer builds meaningful connections and trustworthy relationships that bridge the gap between health and social care, offer hope for a better life, empower people to take an active role in their own care, and achieve their life goals beyond health care. Integrating a peer in a primary care team brings a holistic vision that humanizes care and carers. The peer is a reassuring presence for both the patients and the primary care team, as they help overcome therapeutic impasses by better identifying the patient’s needs and the resources to meet those needs. In doing so, peers contribute to a decrease in mistrust toward the health and social services system, which allows the creation of a bridge between primary care, community resources, and the living environment.

Our findings showed that temporality is one of the most important features and added value of peer support work in a primary care team. Taking the time to listen, to gain trust, to build relationships, to understand needs, and to make connections that bridge health and social care is central to the work of the peer. Yet, it is important to keep in mind that peers do not have more time than primary care clinicians do. It is the nature of peer support work, as it is the case for professionals, to take the time to build relationships by making themselves available when needed without feeling pressured. Building trust, recognizing suffering, and validating emotions and painful experiences takes time, but the results seem more lasting because the causes of suffering are being addressed.

While the research funds facilitated the recruitment of a highly experienced peer, it is crucial to acknowledge that the observed results might not be replicable in resource-limited settings without similar access to experienced peer support workers. Future research should explore the impact of peer support when delivered by peers with varying levels of experience and in settings with differing levels of resources to determine if similar results can be achieved. This could inform policies around peer compensation and sustainability of peer support programs in primary care.

### Participatory analysis and interpretation

Participatory analysis of the results with the primary care providers and the peer brings nuance to their interpretation. Some results were put into context that was not captured by the research interviews. Regarding the perceived effects of peer support, results indicated that the relationship with the peer promotes acceptance and greater understanding of the disease or condition. Though understanding leads to action and empowerment, acceptance leads to passivity. To address this, primary care providers and the peer bring the nuance of fostering acceptance of life with disease but not necessarily acceptance of pain or disease itself. The peer explains that the use of the term “accepting one’s disease or condition” also implies its opposite, “refusing.” Yet, it is not possible to accept or refuse one’s illness or condition; they simply exist and people have to deal with them. This is an important nuance for modulating outcome expectations. That is, peers do not have the goal to promote acceptance of pain and disease but to offer hope for living fully despite them. This is why the distinction between disease, understood as a biological condition, and illness, as a socially constructed meaning of the biological condition, is important to understand the mechanism of peer support intervention in primary care practices.

In addition, participatory analysis has enabled us to deepen our interpretation of temporality. Although the understanding of temporality is set in opposition in our results (the peer has time to listen while clinicians do not), the interpretation of this finding must go beyond this dichotomous vision. Rather, peer support is a complementary approach that emphasizes the role of the peer in overcoming therapeutic impasses and issues of trust between patients and clinicians. Therefore, an innovative feature of peer support in primary care is related to their capacities to repair and forge therapeutic alliances with the most vulnerable people by emphasizing the importance of partnership with patients in overcoming therapeutic impasses. Positioning temporality as a complementary approach focused on the therapeutic alliance reveals one of the greatest added values of integrating a peer into a primary care team.

### Challenges and ethical considerations

Findings on challenges and ethical considerations such as personal information disclosure and managing relationship boundaries in the absence of institutional guidelines and formal status are similar to those reported in the literature [8, 9, 28, 29]. These challenges can act as barriers for other teams wishing to implement a peer support initiative because not understanding the peer support work hinders buy-in and recognition of the added value of integrating a peer into a primary care team. However, feedback from research clarifies the nature of accompaniment and the role of peers in a primary care team, which quickly promotes acceptance of this role in the primary care setting, thus leading to actions to overcome challenges highlighted in our study. The ethical framework was developed to provide support to peers integrating primary care teams by delineating the role and by developing communication mechanisms. Finally, the ethical framework could help overcome barriers related to financial compensation by making the role official without professionalizing it, and support scaling in another primary care setting.

### Limits

This study’s focus on a single peer within a unique primary care setting, with a small sample size of eight patients and their relatives, limiting the generalizability of findings. We stress that, even though it is not the goal of an exploratory study on the feasibility of integrating peers into a primary care team, the results of our study are not generalizable in other contexts or settings. Although they have not been measured, the perceived effects are similar to the impacts identified in the peer literature. For this reason, integrating a less experienced peer could result in effects, challenges, and risks that were not identified or foreseen in our study. Finally, the study took place at the height of the COVID-19 pandemic. Consequently, interviews were conducted on Zoom or by phone, limiting human contact between researchers and participants.

## Conclusion

The study found that peers establish a connection with patients through sharing their own difficult experiences and how they overcame them, rather than sharing similar health or social conditions. Peers provide social support and coaching beyond the care trajectory and can link identified needs with available resources in the community, bridging the gap between health and social care. Primary care clinicians benefit from peer support work, as it helps overcome therapeutic impasses and facilitates communication of needs and issues between patients and their primary care providers. This study clarified how a peer support project unfolded in a primary care setting, and clearly showed the ability of a peer to reach people with complex medical and social needs. Integrating peers in primary care teams is therefore a meaningful way to act on social determinants of health and to address challenges associated with complex health and social needs for both patients and primary care teams. Our findings are consistent with studies on peers in mental health, addiction, and homelessness, as they work towards overcoming obstacles to prevention and care, reducing distrust of institutions, prioritizing needs, and navigating the complexities of healthcare services. Findings showed the added value of integrating peers in a primary care team and setting core principles for other teams wishing to do so. By gaining trust, offering hope, and bridging health and social care, peers embodied integrated community care.

## Data Availability

Availability of data and materials In accordance with ethical standards of the participants’ consent (#2020-564, DIS-1819-77), the data that support the findings of this study are not openly available to protect confidentiality and participant’s identity. Data is located in a controlled access data storage at CRCHUM. Raw de-identified data may be made available upon reasonable request from the corresponding authors.
